# News and Notes

**Published:** 1908-06

**Authors:** 


### NEWS AND NOTES

The German government has prohibited the future use of salicylic acid as a preservative.

---

Since the prohibition of the use of goat's milk, Malta fever has decreased in a remarkable manner in the island of Malta.

---

Unusually severe outbreaks of small pox are reported from Japan, especially in Tokio and Yokohama.

---

According to the report of the Pennsylvania Railroad Employe's Relief Fund for the twenty-five years of its existence, $18,000,000 in benefits has been paid out.

---

Miss Florence Nightingale, who won world-wide fame by her work in the camps and among the military hospitals during the Crimean war, has just been added to its roll of Freeman by the city of London.

---

During the month of March there were 198 deaths in the Canal Zone, including the cities of Panama and Colon, in a population of 114,920, corresponding to an annual death rate of 20.67 in 1,000 of population.

---

Dr. John C. Hemmeter, urges that the monument to be erected to Dr. Walter Reed in Washington, include Drs. Lazear and Carroll, who were also members of the yellow fever commission.

---

The annual meeting of the Georgia State Sociological Society was held at Atlanta, in the Piedmont assembly hall, Monday and Tuesday, the 4th and 5th of May.

---

Dr. J. McF. Gaston and wife have been appointed missionaries to North China by the Foreign Mission Board of the South. Dr. Gaston goes as a medical missionary and will assume charge of the Tyzzar Hospital at Laichon Tu, in the province of Shantung. Dr. Gaston has been practicing medicine in Atlanta for 15 years and has been prominently connected with the Missionary Medical School.

The measure proposed in Virginia to exempt physicians from paying a license fee has been defeated.

---

"The Southern Medical Journal" is the name of a new journal which is to be published at an early date in Nashville, Tenn.

---

A class of thirty-five were graduated from the Medical Department of the University of Georgia, at Augusta, May 1.

---

The Maryland house and senate have passed the so-called anti-Christian Science bill which prohibits all Eddyists from practicing medicine unless they have passed the regular examination, or eschew the acceptance of fees.

---

Mulford & Co., have issued some interesting "Working Bulletins" on vaccine therapy, Wright's opsonic theory and its practical application. The supplement to Bulletin No. 1, furnishes further information on the dosage an dmethod of administration of bacterial vaccines. Bulletin No. 2, is on tuberculin and tuberculin therapy.

---

The Georgia Pharmacutical Association held its meeting in Thomasville, May 19 and 20. J. D. Persse, of Savannah, is president of the Association; L. S. Brigham, of Columbus, is vice president; B. S. Persons, of Macon, and T. B. Rice, Greensboro, vice presidents; J. T. Shuptrine, of Savannah, treasurer, and Max Morris, of Macon, secretary.

---

The California Anti-Narcotic law goes into effect July 1, 1908, and partially prohibits the sale of narcotics without a physician's prescription and states that no poison shall be sold to any person less than eighteen years of age.

---

The fourth annual meeting of this association will be held at the Auditorium Hotel, Chicago, on June 5th and 6th. An extensive program has been arranged, and the meeting promises to be both interesting and profitable.

At the annual meeting of the American Surgical Association, which was held recently in Richmond, Va., the following officers were elected for the ensuing year: President, Dr. C. B. G. Nancrede, of Ann Arbor, Mich.; vice presidents, Dr. A. P. Gerster, of New York, and Dr. Leonard Freeman, of Denver; treasurer, Dr. Charles A. Powers, of Denver; secretary, Dr. Robert G. Leconte, of Philadelphia. The meeting in 1909 will be held in Philadelphia.

---

Dr. Rudolph Matas, 2255 St. Charles avenue, New Orleans, La., is compiling the statistics of operations for the cure of aneurism by his method of intrasaccular suture. (endoaneurismorrhaphy), and will be obliged to all surgeons who have had experience with this operation for a brief report of their cases.

---

The freedom of the City of London has been conferred on Miss Florence Nightingale. At her request the illuminated resolution presenting her with the freedom of the city, was closed in an oaken instead of, according to the ancient custom, golden casket, and a check for 100 guineas was devoted for the furtherance of those objects to which she has given her whole life.

---

The preesident of the German Colonial Society, Duke Johann Albrecht zu Mecklenburg, announces that a prize of $1,500 will be awarded to any one who can suggest a means by which cattle can be protected against the bites of the tsetse flies while they are being taken through a region infested with these flies.

---

The alumni of the Medical Department of the Tulane University of Louisiana propose to give a Stanford E. Chaille jubilee on May 19 and 20, to celebrate the completion of the fiftieth year of his service as dean of the medical department. Professor Chaille retires from the faculty and deanship at the end of the current year. It is proposed to establish a Chaille Memorial Fund from the alumni and friends of the Tulane University, the funds obtained to be used for a dormitory for medical students and the revenue derived to be used for the support of a chair of physiology or hygiene, to be named after Dr. Chaille. It is hoped that a subscription of $15,000 or $20,000 will be made for this purpose.

### TO HONOR DR. CHAILLE.

We note with pleasure the plan of the Alumni of the Medical Department of Tulane University to celebrate the fiftieth year of teaching service of Professor Chaille. The celebration is to take place on May 19 and 20, 1908 in the form of a jubilee.

It is further proposed that a fund be created to preserve the memory of Dr. Chaille and his highly appreciated services to the institution. The Alumni and friends of Dr. Chaille are requested to send their contributions to Dr. Isadore, secretary and treasurer of the Chaille Memorial Fund, P. O. Box 778, New Orleans.

We believe there is no man in the South to whom greater honor is due than to Dr. Chaille and his remarkable service at Tulane.

---

If a patient persists in running evening temperatures which cannot be accounted for after a thorough physical examination and blood examination, one should place the patient on increasing doses of the iodids, for the fever may be due to an old syphilitic infection.—American Journal of Surgery.

---

In cases of suspected fracture of the skull, percussion-auscultation will be found a valuable procedure where all the other signs and symptoms have been negative. The procedure is the following: The forehead is repeatedly tapped sharply in the median line with the middle finger, the stethoscope being moved from one point to another from before backward. If a fracture be present, a cracked-pot sound is elicited just beyond it. The corresponding part of the head on the other side should be auscultated to eliminate possible error.American Journal of Surgery.

---

The United States Civil Service Commission anonunces an examination on July 1, 1908, to secure eligibles from which to make certification to fill a vacancy in the position of technical assistant, Division of Pharmacology, Mygienic Laboratory, Public Health and Marine-Hospital Service, at $150 per month, and vacancies requiring similar qualifications as they may occur in any branch of the service

Persistent, remittent fever after an acute infection of the knee joint is usually due to a systemic invasion. Such cases are best treated by laying the joint wide open (Mayo operation).—American Journal of Surgery.

---

### REGULAR MEETING, FULTON COUNTY MEDICAL SOCIETY, MAY 7, 1908.

EDITED BY R. R. DALLY, M. D.

---

DR. STIRLING IN CHAIR.

---

Dr. J. H. Johns read a paper upon Ingrowing Toenail and operation for same. He described a modification of the usual operations wherein he made a wider flap at the base of the nail giving clearer view of the matrix and parts to be removed and insuring completion of just what the operator desired to accomplish. His cases under this method have all been successful.

Dr. Armstrong discussed the paper favorably saying he had seen Dr. Johns operate and had accepted the surgical technique as of great value.

Dr. Armstrong exhibited a kidney showing extensive multilocular cystic degeneration. It was removed from a negro who had suffered many symptoms of diseased kidney, but who had been passing 18 ounces of urine a day. The exploratory operation showed the left kidney to be so greatly diseased that those present believed it could be of no value and they advised removal.

After operation there was anuria followed by the discharge of about 15 ounces and again succeeded by anuria and uremia of which patient died. No postmortem was permitted. so the condition of the other kidney could not be known.

Dr. Strickler remarked that many English surgeons were now making their exploratory incisions in the abdomen and thus having opportunity to examine both kidneys before removing one. He also pointed out that Morris had drained some of these cystic kidneys with at least temporary advantage.

Dr. Kime related a case of infected kidney that was by some surgeons, suspected of being enlarged gall bladder. He made several careful urine examinations, segregating the specimens till he was satisfied that he had pus from the right and urine from the left.

He then operated in the usual region and successfully removed the right kidney. The woman recovered. He believes this is better procedure than making the abdominal incision first and then having to subject the patient to the further strain of the lumbar operation.

Dr. Gaillard spoke in favor of segregation of the specimens.

Dr. Hodgson reported a cases of broncho pneumonia beginning 36 hours after birth. The labor was slow and he suspects the child may have breathed in some infections material during delivery.

At the onset the respirations were very rapid—over a hundred—and the cause was sought in the heavy vitiated air of the room. The child was placed between two open windows and the respirations dropped. Once after that the family allowed the air to get bad, fearing cold, and there was greatly excited breathing. Treatment was continued with fresh air constantly blowing across bed and child recovered.

---

At the annual meeting of the Washington Osteopathic Association, held in Seattle, a candidate for governor was nominated, the association claiming that this was the only one of the various candidates who would give osteopathy a square deal. The association is said to be prepared to expend funds in its treasury to work against any candidate who is known to favor legislation against osteopathy, and to aid in legislation favorable to their cult.

---

A large, slowly healing superficial ulcer of the leg may be due to a thrombosis of one of the small vessels leading to that part. Of course, syphilitic etiology must first be ruled out.—American Journal of Surgery.

